# Important Functions and Molecular Mechanisms of Mitochondrial Redox Signaling in Pulmonary Hypertension

**DOI:** 10.3390/antiox11030473

**Published:** 2022-02-28

**Authors:** Jorge Reyes-García, Abril Carbajal-García, Annarita Di Mise, Yun-Min Zheng, Xiangdong Wang, Yong-Xiao Wang

**Affiliations:** 1 Department of Molecular & Cellular Physiology, Albany Medical College, Albany, NY 12208, USA; reyes.garcia.jorge@gmail.com; 2 Department of Pharmacology, Faculty of Medicine, National Autonomous University of Mexico, Ciudad de Mexico 04519, Mexico; carbajalabril@gmail.com; 3 Department of Biosciences, Biotechnologies and Biopharmaceutics, University of Bari, 4-70125 Bari, Italy; 4 Department of Pulmonary and Critical Care Medicine, Shanghai Engineering Research for AI Technology for Cardiopulmonary, Shanghai 200032, China; 5 Shanghai Institute of Clinical Bio-Informatics, Zhongshan Hospital, Fudan University, Shanghai 200032, China; 6 Jinshan Hospital Centre for Tu-mor Diagnosis and Therapy Fudan University, Shanghai 200540, China

**Keywords:** pulmonary hypertension, mitochondrial ROS, ketones, Ca^2+^ signaling

## Abstract

Mitochondria are important organelles that act as a primary site to produce reactive oxygen species (ROS). Additionally, mitochondria play a pivotal role in the regulation of Ca^2+^ signaling, fatty acid oxidation, and ketone synthesis. Dysfunction of these signaling molecules leads to the development of pulmonary hypertension (PH), atherosclerosis, and other vascular diseases. Features of PH include vasoconstriction and pulmonary artery (PA) remodeling, which can result from abnormal proliferation, apoptosis, and migration of PA smooth muscle cells (PASMCs). These responses are mediated by increased Rieske iron–sulfur protein (RISP)-dependent mitochondrial ROS production and increased mitochondrial Ca^2+^ levels. Mitochondrial ROS and Ca^2+^ can both synergistically activate nuclear factor κB (NF-κB) to trigger inflammatory responses leading to PH, right ventricular failure, and death. Evidence suggests that increased mitochondrial ROS and Ca^2+^ signaling leads to abnormal synthesis of ketones, which play a critical role in the development of PH. In this review, we discuss some of the recent findings on the important interactive role and molecular mechanisms of mitochondrial ROS and Ca^2+^ in the development and progression of PH. We also address the contributions of NF-κB-dependent inflammatory responses and ketone-mediated oxidative stress due to abnormal regulation of mitochondrial ROS and Ca^2+^ signaling in PH.

## 1. Introduction

Mitochondria are important organelles that contribute to cellular homeostasis; thus, the dysregulation of mitochondrial function can lead to cellular or tissue injury, and further systemic affections [1]. Mitochondria are best known for being the powerhouses of aerobic cells. Producing adenosine triphosphate (ATP) through oxidative phosphorylation (OXPHOS) [2] is not the only function of these organelles, yet they are involved in the regulation of Ca^2+^ signaling, redox potential, and the control of reactive oxygen species (ROS) production and levels [3]. 

ROS are important byproducts of O_2_ metabolism occurring in the environment of all cells. Major sources of these species are mitochondria and cytoplasmic enzymes such as nicotinamide adenine dinucleotide phosphate (NADPH) oxidase (NOX) [4]. For a long time, mitochondrial O_2_ derivatives were considered harmful cellular chemical entities; nevertheless, there is increasing evidence showing that ROS are not only toxic oxidants but serve as signaling molecules implicated in several processes, such as cytosolic Ca^2+^ signaling [5,6,7,8,9], gene transcription [10] and protein synthesis [11]. The impairment of mitochondria function (mitochondrial dysfunction) affects almost any functional tissue in the body, e.g., pancreas, skeletal, cardiac and smooth muscle, nerves, kidney, and lungs among others [12]. Mitochondrial dysfunction due to alterations in ROS production and mitochondrial DNA (mitDNA) damage underlies critical pathophysiological mechanisms in numerous diseases such as diabetes [13,14], fibromyalgia [15], chronic heart failure [16], Alzheimer’s disease [17], chronic kidney disease [18], atherosclerosis [19], and pulmonary hypertension (PH) [5,20]. In this regard, the role of ROS has been extensively investigated in vascular biology. The production of ROS is highly increased in PH experimental models and clinical hypertension, and more specifically, the evidence highlights that fluctuations in intracellular ROS concentration ([ROS]_i_), mostly elicited by mitochondrial dysfunction in pulmonary artery (PA) endothelial cells (PAECs) and PA smooth muscle cells (PASMCs), contribute to the progression of PH [6,7,8,9,21,22,23].

In addition to the generation of ATP and ROS, mitochondria are also involved in amino acid metabolism, release of tricarboxylic acid (TCA) cycle metabolites, fatty acid oxidation (FAO) and ketone bodies synthesis [24,25]. Ketone bodies, including β-hydroxybutyrate (β-HB), acetoacetate and acetone, are primarily produced by β-oxidation of fatty acids (FA) in the mitochondria of hepatocytes, even though enterocytes, astrocytes and kidney epithelial cells may produce them as well [24,26]. Interestingly, it has been shown that β-HB decreases the senescence of vascular cells [27], and that ketones abolish the generation of mitochondrial ROS [28]. This review focuses on the participation of mitochondrial ROS (mitROS) and Ca^2+^ signaling in the inflammatory process leading to PH development, as well as their mutual interactions, and the possible role of ketone bodies on mitochondrial Ca^2+^ and/or ROS signaling.

## 2. Pulmonary Hypertension

PH is a rare and fatal disease with an estimated prevalence of 5 to 50 cases per million individuals [29,30,31,32]. This ailment has been defined by the increase in the mean pulmonary arterial pressure (mPAP) ≥25 mm Hg at rest or ≥30 mm Hg during/after workout [33]. Most patients suffering from PH are women, covering around 60% to 80% of all cases [30,34,35]. Therefore, female sex has been considered a risk factor for the development of PH. Nevertheless, male sex has been associated with poorer survival rates and this could be explained by the fact that men affected by this illness exhibit a low right ventricular function recovery [36,37]. The latest update in 2013 of World Health Organization’s clinical classification system has catalogued PH into five categories/groups depending on the main underlying cause, hemodynamics, clinical features, and therapeutic responsiveness [21,33]. As shown in Table 1, Group I refers to pulmonary arterial hypertension (PAH) and encloses idiopathic PH (IPAH), drug-induced PH, heritable PH, and PH associated with other systemic diseases. PH due to left heart disease corresponds to group II. Groups III and IV include PH due to lung diseases and PA obstructions, respectively. Lastly, group V describes PH with unclear multifactorial mechanisms. Apparently, a number of pathological causes may cause and promote dysfunctions of PA endothelial cells and PASMCs via numerous distinctive signaling mechanisms, thereby leading to the initiation and development of PH, right ventricular failure (RVF) and even death [21,33]. 

The pathophysiology of PH involves continuous pulmonary vasoconstriction, endothelial cells (ECs) injury, vascular smooth muscle (VSM) damage and proliferation, intimal fibrosis, remodeling, and inflammation. These alterations, i.e., pulmonary vascular remodeling (PVR), promote an augmented pulmonary vascular resistance (by the occlusion of blood vessels), increasing the right ventricle afterload and leading to right ventricular hypertrophy and failure, and eventually death [38,39]. The enhanced pulmonary vascular reactivity is also associated with malfunctioning of endothelial cells, leading to an imbalance in the production of nitric oxide (NO), prostaglandin (PG)-I_2_ (also named prostacyclin), and endothelin-1. More specifically, it has been shown that patients suffering from PAH display reduced expression of endothelial nitric oxide synthase (eNOS) and NO levels in the lungs [40,41]. In addition, the expression of prostacyclin synthase is diminished in patients with severe PH [42]. Importantly, PGI_2_ triggers the synthesis of cyclic adenosine monophosphate (cAMP) and stimulates the peroxisome proliferator activated receptor-γ (PPARγ), leading to an antiproliferative effect in VSM cells (VSMCs) [43]. Eventually, this endothelial perturbance results in a diminished endothelium dependent pulmonary vasculature relaxation [44,45]. 

Contractile VSM mechanisms are also modified during PH. For instance, the expressions of RhoA and Rho-associated protein kinase (ROCK), important elements of Ca^2+^ sensitization, are elevated in a mouse model of PH [46]. Likewise, Ca^2+^-activated K^+^ (K_Ca_^2+^) channel 3.1 subtype is increased during PH contributing to VSM proliferation and remodeling [47]. On the other hand, the activity of voltage-gated potassium (K_V_) channels is lessened [48], leading to persistent PAs contraction. PVR is caused by hypertrophy and hyperplasia of VSMCs, loss of small pre-capillary arteries, neointimal formation, adventitial thickening and plexiform lesions due to disturbances in the apoptosis and proliferation of VSMCs and ECs [49]. Muscularization and wall thickening of peripheral pulmonary arteries set the basis for the increased vascular resistance and persistent contraction seen in PH. Furthermore, the development of this disease has been related to pro-inflammatory or infectious etiologies such as scleroderma, human immunodeficiency virus (HIV), and schistosomiasis which often have systemic vascular complications [50,51,52]. Researchers have established animal models of PH using monocrotaline injection (MCT), chronic hypoxia, and Sugen 5416 (a vascular endothelial growth factor inhibitor) with chronic hypoxia. These three animal models, which replicate key features of PH in humans, have been assessed in intact animals, PAs, PASMCs and PAECs. The findings have led to significant insights into the development, mechanisms, diagnosis, and treatments of PH [53,54,55,56]. 

During heart failure, a shift from FAO-based metabolism to glycolysis occurs. Under normal circumstances, the ATP produced in cardiomyocytes is primarily generated from FAO; nevertheless, during stress conditions, such as an enhanced ROS environment, FAO may be reduced as glycolysis increases [57]. Metabolism of FA and ketones predominates in patients suffering from PAH with a low glucose control [58].

## 3. Inflammation in Pulmonary Hypertension

The inflammatory response is implicated in the development of PH, particularly in PAH subtype due to diverse molecular pathologies [59]. Perivascular inflammation precedes pulmonary vascular lesions. Endothelial injury favors the participation of chemokines involved in the recruitment of inflammatory cells and intravascular infiltration. Chemokine (C-X3-C motif) ligand 1 (CX3CL1/Fractalkine) serves as a cell adhesion molecule that acts through its own receptor (CX3CR1) and promotes the recruitment of monocytes, dendritic cells (DCs), mast cells, and subpopulations of T-cells [60,61]. In this regard, distinct chemokines have been found to be increased in PAH patients. For instance, CX3CL1 is upregulated in circulating CD4+ and CD8+ T-lymphocytes [60]. Chemokine (C-C motif) ligand (CCL) 5 (CCL5/RANTES) is also augmented [62], and is responsible for regulating the activation of T-cells and neutrophils [63,64]. Moreover, CCL2/MCP-1 was found to be elevated in patients with PH and activates macrophages to induce the expression and secretion of adhesion molecules and other cytokines [65]. In addition, CX3CR1 deficiency reduces monocyte recruitment and macrophage polarization in hypoxia-induced PH [66]. Circulating inflammatory cells, such as DCs and mast cells, recruited by the above-mentioned chemokines are directed to sites of endothelial injury. In IPAH patients, vascular lesions exhibit immature DCs infiltration [67]. DCs may be implicated in the presentation of antibodies against endothelial cells, fibroblasts, and naive T-cells. Infiltration of T lymphocytes, and particularly CD3+ and CD8+ T cells, has been observed in the lungs of patients with PAH [68,69]. Furthermore, regulatory T cells (Treg) can modulate the endothelial function of the pulmonary artery, inflammation, and SMC proliferation [70]. Studies have shown that patients affected by IPAH present a higher proportion of circulating Treg than healthy subjects [71]. Although mast cells mainly play an essential role in allergic inflammation, these cells are also found to be increased in patients with PH [72]. Mast cells degranulation release interleukin (IL)-4, which stimulates B cells to secrete anti-endothelial cell antibodies contributing to hypoxic PVR and PH [73]. 

Cytokines released from recruited inflammatory cells mediate communication between endothelial and other vascular cells, e.g., VSMCs. In PH, increased levels of IL-1, IL-6 and tumor necrosis factor (TNF)-α are exhibited [74,75,76]. It has been suggested that the heightened levels of IL-1 and IL-6 derives primarily from lung microvascular endothelium [77]. In a PH rat model induced with MCT, excessive amounts of IL-1 were found in the lungs [78]. Furthermore, elevated levels of IL-6 have been related with poor survival in patients with PH [79]. All these inflammatory alterations result in vascular remodeling, leading to increased pulmonary vascular pressures and resistance. In this context, mitochondria undergo physiological and structural changes during PH [80], and correspondingly ROS levels are altered [81,82]. 

Nuclear Factor κB (NF-κB) is a master regulator of inflammation implicated in the development of PH. NF-κB is a family of inducible transcription factors considered as the main controllers of innate immunity [83]. Five structurally related family subunits have been identified: p50, p51, RelA (p65), RelB, and c-Rel. The activation of these family members depends on the degradation of the inhibitor of NF-κB proteins (IκBs), which hold inactive NF-κB dimers in the cytosol upon the stimulation of determined cells.

Two major signaling pathways have been described for NF-κB. The canonical pathway involves the release of the p65/p50 subunits from the IκB complex, promoting the translocation of the heterodimer to the nucleus [84]. The activation of the canonical NF-κB signaling cascade induces the expression of several genes encoding pro-inflammatory cytokines and chemokines, including TNF-α, IL-1β, IL-6 and other inflammatory mediators, such as anti-apoptotic factors, cell cycle regulators, and adhesion molecules [85,86,87]. On the other hand, the noncanonical (alternative) NF-κB pathway does not require the degradation of inhibitory IκB complex, but the processing of p100, the protein precursor of p65 [85]. 

Regarding the role of NF-κB in PH, Sawada et al. in 2007 reported that the stimulation of NF-κB, leading to the activation of the vascular cell adhesion molecule (VCAM)-1, is related to the development of MCT-induced PH in rats. Moreover, the use of the NF-κB inhibitor, pyrrolidine dithiocarbamate (PDTC), decreases PH symptoms [88]. Later, Huang et al. using the same animal model demonstrated that PDTC restores endothelial cell membrane integrity by rescuing caveolin-1, leading to PH [89]. Likewise, the inhibition of NF-κB with PDTC has proven to be effective in decreasing arterial lumen obliteration in SU5416-induced PH [90]. A novelty technology implemented by Kimura et al., based on a decoy directed against the NF-κB binding site in the promoter region, attenuated inflammation, proliferation, and pulmonary artery remodeling in rats. The implemented nanotechnology may serve as an advanced molecular approach for the treatment of PAH patients [91]. Moreover, Hosokawa et al. demonstrated that IMD-0354, a NF-κB inhibitor, blocks p65 translocation to the nucleus and decreases the proliferation of PASMCs associated with PH [92]. 

Interestingly, the role of NF-κB in a hypoxia-induced PH model has been investigated as well. It is well known that chronic hypoxia can lead to apoptosis, vascular remodeling and ultimately PH [93]. Hypoxia-inducible factor (HIF)-1α augments its transcriptional activity in response to oxygen decline in the lung [94]. In this context, Luo et al. demonstrated that NF-κB mediates the transcriptional program of HIF-1α promoting vascular remodeling in a PH model [95]. Eventually, it has been shown that the abnormal activity and regulation of NF-κB exacerbate the inflammatory and Ca^2+^ responses in PASMCs from PAH patients [96]. 

## 4. Mitochondria in Vascular Remodeling during PH

The mitochondria of the vascular smooth muscle cells (VSMCs) and PAECs, as in any other cells, are responsible for the synthesis of ATP, the key energetic molecule, thus a strict control of metabolism is exerted by these double-membrane-bound organelles [97]. In addition, mitochondria take an important place in the production and regulation of ROS, Ca^2+^ signaling, metabolism of glucose and FA, and apoptosis. These mechanisms are essential players in the development of PVR seen in PH disease [49]. During PH, a disfunction in mitochondria’s metabolism occurs, particularly a shift in energy production from OXPHOS to glycolysis and lactic acid fermentation in order to maintain ATP production and cell survival [98]. This phenomenon, known as the Warburg effect, was described in 1956 by Otto Warburg in tumor cells under normal oxygen conditions to support the uncontrolled growth of neoplastic tissue [99,100]. Hyperproliferation, survival and metabolic reprogramming of PASMCs and PAECs set the basis for the pathophysiology of PH [101,102]. Moreover, mitochondria in PASMCs sense the oxygen levels, and patients suffering from PH display abnormalities in this mechanism [103]. Furthermore, PVR involving structural changes in intima, media and adventitia is linked to a marked inflammatory process in pulmonary hypertension [49,104]. However, the precise mechanisms underlying this relationship are still uncertain and mitochondrial dysfunction may serve as an explanation. 

Alterations in mitochondrial respiration can lead to variations in mitochondrial membrane potential (MMP) [105]. MMP has shown to be either hyperpolarized [106,107,108] or depolarized [109] in PH models. Mitochondrial uncoupling proteins (UCPs) participate in the control and regulation of MMP and ROS production. Five UCP homologues have been characterized in mammals (UCP1-UCP5) [110]. In this regard, Pak et al. showed that the genetic ablation of UCP2 promotes the proliferation of PASMCs in mice. Additionally, they found that MMP and ROS production are increased in PASMCs from patients and in animal models of MCT- and hypoxia-induced PH [80]. Recently, it has been shown that heat-shock protein 90 (HSP90), in response to stress, accumulates in the mitochondria of PASMCs from PH patients to protect mitochondrial DNA (mitDNA) and preserve mitochondrial functions leading to cell survival. Moreover, the inhibition of mitochondrial HSP90 (mtHSP90) diminishes mitDNA content, restores mitochondrial bioenergetics and limits the hyperproliferative state of PASMCs [111]. 

As well as mitochondrial dysfunction, endoplasmic reticulum (ER) stress is implicated in PH pathophysiology, and an interesting interplay between mitochondrial and ER stress drives some aspects of this disease. For instance, the loss of function of bone morphogenetic protein receptor type II (BMPRII) has been shown to induce ER stress, being a critical genetic factor predisposing to PAH [112]. Restoring BMPRII performance and the abolishment of ER stress have been remarkable suggested as a potential treatment against PH [113,114]. Mitochondrial fragmentation complemented with ER stress have been observed in PASMCs from hypoxic-induced PH rats [115]. Moreover, the same work exhibits that mitochondrial fragmentation promotes ER stress through a ROS dependent mechanism and the abolishment of ER stress improves PASMCs function under hypoxic condition. More interestingly, the mitochondrial division inhibitor (Mdvi-1) decreases mitochondrial fragmentation, ER stress and improves PASMC performance [115]. It is known that ER stress can cause unfolded proteins to accumulate in the ER and then activate the unfolded protein response (UPR). The persistent UPR conduces to the dysfunction of mitochondria accompanied by the disturbance of mitochondria-associated ER membrane (MAM), ultimately leading to cell apoptosis [116,117]. In this context, it has been observed that S-nitroso-L-cysteine (CSNO), a derivative of NO, improves ER stress and regulates the expression of contractile smooth muscle proteins in the lungs of MCT-induced PH rats. CSNO also leads to smooth muscle relaxation via anti-inflammatory pathways, ameliorating PVR [22]. Furthermore, the disruption of MAMs diminishes mitochondrial aberrations in ECs under hypoxic stimulus through an augment in NO release and the inhibition of the proinflammatory profile induced by hypoxia [118]. Lastly, mitochondria and ER morphology and dysfunctions in PASMCs and PAECs arise as novel potential therapeutic targets for the treatment of PH, although further research is needed. 

## 5. Mitochondrial ROS in Pulmonary Vasoconstriction and Endothelial Dysfunction

Variations in [ROS]i in pulmonary vascular cells play a role in the pathogenesis of PH. Oxidative stress and ROS signaling in PASMCs are involved in PA vasoconstriction and remodeling and, therefore in the development and progression of PH [119]. It is well established that mitochondria account for the most production of ROS in PASMCs [9,120]. Mitochondria are considered an important factor in PVR due to their participation in numerous proliferative signaling pathways, such as regulating ROS production, ATP balance, apoptosis, metabolism of glucose and FA, or controlling Ca^2+^ homeostasis. In particular, ROS produced by mitochondrial complexes I, II and III have been suggested to play an important role in the development of PH. For instance, genetic deletion of the core subunit of mitochondrial complex I, NADH dehydrogenase (ubiquinone) iron–sulfur protein 2 (NDUFS2), was reported to decrease mitROS (H_2_O_2_) production and abolish hypoxia-induced pulmonary vasoconstriction (HPV) in mice and rats [121]. In this context, HPV is known to redirect blood flow from hypoxic to better ventilated areas of the lung. Moreover, HPV and arterial occlusion are important causes of PH as they decrease blood flow and increase vascular resistance. HPV appears to be mediated in part by an increase in intracellular Ca^2+^ and ROS signaling. [122]. In addition, Paddenberg et al. showed that Succinate dehydrogenase (ubiquinone) cytochrome b small subunit, (SDHD), is necessary for optimal functioning of the mitochondrial complex II and for HPV [123]. Furthermore, the mitochondrial complex III, and in particular the Rieske iron–sulfur protein (RISP), has shown to be required for mitochondrial ROS production in PASMCs [7,124,125]. Interestingly, our research group has shown that RISP is essential for the development of chronic hypoxia-induced PH. Knockdown of this sulfur protein in vivo reduces HPV and abolishes the hypoxia-induced increase in right ventricular pressure and the increase in right ventricular weight [5]. The balance between mitochondrial fusion and fission is essential for the physiology of this organelle. The role of dynamin-related protein 1 (DRP1) in mitochondrial fission may contribute to the disruption of ECs function and hyperproliferation of VSMCs involved not only in PH. The aggressive ROS environment triggers DRP1 signaling and mitochondrial fission, eliciting the enhancement of ROS production (ROS-induced ROS generation) [126]. On the other hand, the nuclear factor erythroid 2-related factor 2 (Nrf2) is one of the systems involved in the regulation of antioxidant genes and mitochondrial fission. In this regard, it has been shown that the activation of Nrf2 prevents PVR by blocking endothelial-to-mesenchymal transition [127]. 

## 6. Mitochondrial Ca^2+^, ROS, and Glutaminolysis 

The homeostasis of intracellular Ca^2+^ concentration ([Ca^2+^]_i_) is crucial to maintain the vascular tone. At rest, basal [Ca^2+^]_i_ is tightly regulated to be around 100 nM. After cellular stimulation with a vasoconstrictor agonist, such as norepinephrine, endothelin, vasopressin, etc., [Ca^2+^]_i_ increases reaching values between 500 nM and 1 mM [128]. 

**Figure 1:**
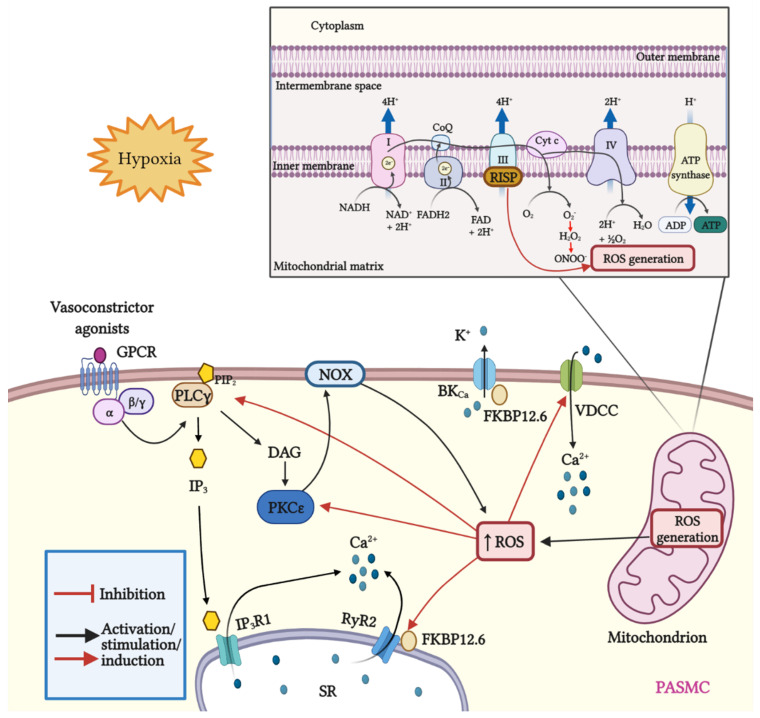
Schematic representation of mitROS generation and signaling; crosstalk between ROS and Ca^2+^ signaling in PASMCs. Mitochondria are the major source of ROS in pulmonary artery smooth muscle cells (PASMCs). During ATP synthesis in the electron transport chain (ETC), coupling between the proton gradient on either side of the inner mitochondrial membrane leads to the production of ROS. Briefly, electrons are transferred from nicotinamide adenine dinucleotide (NADH) and flavin adenine dinucleotide (FADH2) to molecular oxygen. In this process, protons are pumped from the mitochondrial matrix into the intermembrane space, and oxygen is reduced to H_2_O. Hypoxia increases the production of mitROS, contributing to the increase in [Ca^2+^]_i_ and to hypoxia-induced pulmonary vasoconstriction. The Rieske iron–sulfur protein (RISP), a catalytic subunit of the complex III of the mitochondrial ETC serves as a primary molecule in intracellular ROS generation in PASMCs, especially under hypoxic conditions. In addition, mitROS and vasoconstrictor agonists stimulate the PLCγ and PKCε signaling pathways via GPCR activation. PLCγ induces the formation of IP_3_ and DAG, causing the opening of IP_3_R1 and the release of Ca^2+^ from the sarcoplasmic reticulum (SR). Moreover, mitROS augment the activity of PKCε, which in turn stimulates NOX and promotes the formation of ROS in a process named ROS-induced ROS generation (RIRG). In addition, ROS enable the dissociation of FK506 binding protein 12.6 (FKBP12.6) from ryanodine receptor 2 (RyR2) favoring the opening of this channel and enhancing Ca^2+^ release. Furthermore, FKBP12.6 is physically bound to high conductance K^+^ channels (BK_Ca_) and regulates their open probability. Finally, ROS upregulate voltage-dependent Ca^2+^ channels (VDCCs) which further contribute to the increase in [Ca^2+^]_i_, leading to persistent vasoconstriction observed in PH.

Dr. Wang’s research group and other investigators have demonstrated that ROS facilitates the dissociation of FKBP12.6 from ryanodine receptor 2 (RyR2) to activate the channel [125,129,130,131]. Moreover, we have demonstrated that Rieske iron–sulfur protein (RISP) knockdown (KD) abolishes the hypoxic ROS formation in isolated PASMCs, whereas RISP overexpression produces the opposite effect. RISP KD also inhibits the hypoxic increase in [Ca^2+^]_i_ in PASMCs [124,132]. Most recently, we showed that the dissociation of the FKBP12.6/RyR2 complex (induced by chronic hypoxia) causes sarcoplasmic reticulum (SR) Ca^2+^ leak and increases [Ca^2+^]_i_ in PASMCs (Figure 1), thereby leading to subsequent pulmonary artery remodeling and vasoconstriction. These events may occur due to the mitochondrial RISP-dependent ROS generation and the subsequent RyR2 oxidation [5,9]. Furthermore, FKBP12.6 is also specifically bound to big-conductance Ca^2+^-activated K^+^ (BK_Ca_) channels in VSMCs. These channels as well as RyRs are essential proteins in mediating vascular smooth muscle tone.

Mitochondrial Ca^2+^ uniporter (MCU) regulates mitochondrial Ca^2+^ (mitCa^2+^) allowing Ca^2+^ uptake. We have reported that Ca^2+^ release mediated by hypoxic or RyR simulation evokes an improved performance in the activity of MCU. The increased MCU leads to the generation of mitROS dependent on ROS, provoking a positive feedback mechanism to potentiate hypoxia-initiated mitROS in PASMCs [9]. This finding indicates an important role of mitROS and MCU in HPV and associated PH. Alterations in ROS production can alter the physiology of ion channels in PASMCs and induce a large increase in [Ca^2+^]i [133]. Mitochondrial ROS production (after hypoxia) block voltage dependent K^+^ (K_V_) channels [134]. Expression of K_V_1.2, K_V_1.5, and K_V_2.1 channels is reduced in human and animal models of PH [135,136] and in PASMCs following a hypoxic stimulus [137]. Inhibition of these channels causes membrane depolarization and opening of voltage dependent Ca^2+^ channels (VDCCs) with a subsequent large increase in [Ca^2+^]i and vasoconstriction [138]. 

**Figure 2:**
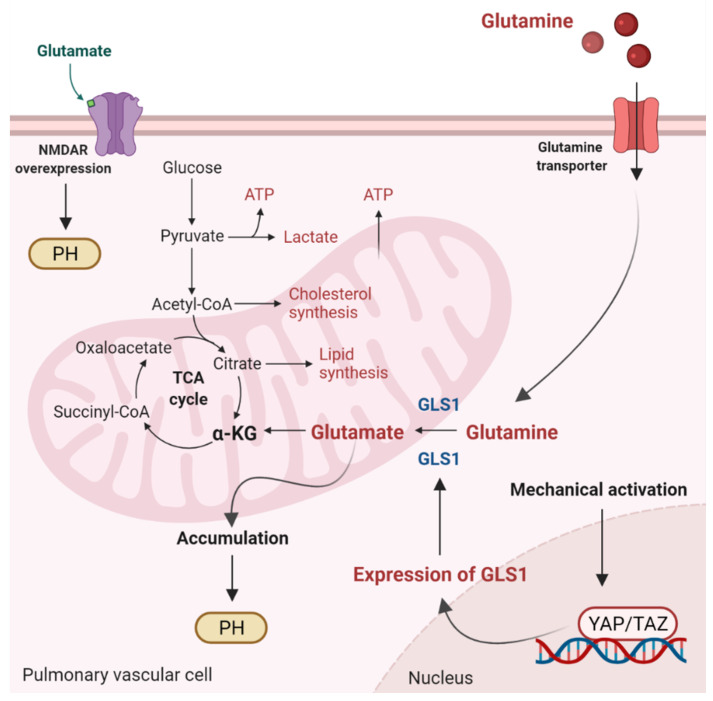
Glutaminolysis and glutamate accumulation contribute to pulmonary hypertension (PH). Glutaminolysis is a mitochondrial process responsible for obtaining cellular energy from the breakdown of glutamine. In this cellular pathway, glutamine is converted into glutamate, aspartate, CO_2_, pyruvate, lactate, alanine, and citrate. Initially, glutamine enters the pulmonary vascular cells via a glutamine transporter and is deaminated to glutamate by glutaminase (GLS1). Subsequently, glutamate is converted to α-ketoglutarate (α-KG) by glutamate dehydrogenase. α kg enters the tricarboxylic acid (TCA) cycle, where it is decarboxylated by α kg dehydrogenase to succinyl-CoA and CO_2_, providing energy for proliferating cells. Accumulation of glutamate in pulmonary vascular cells promotes PH. In addition, stiffening of the extracellular matrix in remodeled pulmonary cells activates the transcriptional coactivators Yes-associated protein 1 (YAP) and TAZ, leading to upregulation of GLS1 and enhanced glutaminolysis. Furthermore, in remodeled pulmonary arteries, the N-methyl-d-aspartate receptor (NMDAR) is overexpressed and overactivated.

Glutaminolysis is a mitochondrial process responsible for obtaining cellular energy from the deamination of glutamine to glutamate by glutaminase (GLS1) [139]. Subsequently, glutamate is converted to α-ketoglutarate (α-KG) by glutamate dehydrogenase. This process (anaplerotic reactions) helps to replenish the intermediates of the TCA cycle after they have been consumed and provides energy especially for proliferating cells. The increase in glutaminolysis leads to increased expression of GLS1 and increased uptake of glutamine by the pulmonary vasculature, resulting in increased glutamate production by pulmonary vascular cells and promoting PH. In addition to glutamate accumulation, the N-methyl-d-aspartate receptor (NMDAR) is overexpressed and overactivated in remodeled pulmonary arteries [140]. Moreover, stiffening of the extracellular matrix of vessels directly regulates glutaminolysis via mechanical activation of Yes-associated protein 1 (YAP) and TAZ. Activation of the former transcriptional coactivators triggers upregulation of GLS1 and leads to glutaminolysis, which maintains the hyperproliferative state and migration of pulmonary vascular cells in PH (Figure 2) [141]. As for PH, the relationship between ROS and glutaminolysis has not been studied. However, glutaminolysis has been shown to trigger the formation of ROS and make hyperproliferative cells (cancer cells) sensitive to ROS [142]. Whether glutaminolysis leads to the formation of ROS in pulmonary vascular cells is unknown and represents an interesting area of research.

## 7. Ketones and Mitochondrial Signaling 

Ketone bodies, or simply ketones, are highly polar molecules produced by β-oxidation of fatty acids in the mitochondria of the liver cells. However, these molecules may be produced by enterocytes, astrocytes, and kidney ECs to a lesser extent [24,26,143]. Ketones are produced in response to reduced glucose availability, e.g., during periods of prolonged fasting, high-performance exercise, or a pathophysiological state, such as type I diabetes [144,145]. It has been postulated that patients with PAH have reduced oral glucose tolerance and lipid and ketone metabolism predominate over the glucose control [58]. 

Ketone’s metabolism is divided in ketogenesis and ketolysis. Ketogenesis, which mainly occurs in perivenous hepatocytes, produces three molecules: acetone, acetoacetate, and β-HB [146], which represents the most abundant ketone body [147,148,149]. It is well known that adipocytes store great amounts of energy as fatty acids [150]. When fasting or exercising, glycogen stores are used in the beginning. Once glycogen is depleted, fatty acids from adipocytes are transferred into the liver by the enzyme carnitine palmitoyltransferase (CPT-1), where they are metabolized in the mitochondria to form ketone bodies [151,152]. These lipid derivatives enter the systemic circulation and reach highly metabolic tissues, e.g., muscles and nervous system, which convert ketones into acetyl coenzyme A (acetyl-CoA) for alternative energy metabolism. The detailed process occurs as follows: two molecules of acetyl-CoA are biotransformed in acetoacetyl-CoA by the action of the acetyl-CoA acetyltransferase (ACAT), a thiolase [153]. Then, 3-hydroxy-3-methylglutaryl Co-A synthase (HMGS2) condenses acetyl-CoA with acetoacetyl-CoA to produce HMG-CoA. Afterward, HMG-CoA is broken into acetoacetate via HMG-CoA lyase (HMGCL). Finally, acetoacetate is further bioconverted to acetone (by decarboxylation) or to β-HB by the action of 3-hydroxybutyrate dehydrogenase (BDH1) [148]. 

On the other hand, throughout ketolysis, acetoacetate and β-HB are used as an energy source by the mitochondria of several extrahepatic tissues [148,154]. Β-HB is transformed to acetoacetate by the BDH1, and this last product is turned into acetoacetyl-CoA by the enzyme beta-ketoacyl-CoA transferase (OXCT1 or SCOT). Acetoacetyl-CoA is separated by thiolase in two molecules of acetyl-CoA [155], that enters the TCA, and subsequently the oxidative phosphorylation, resulting in the generation of ATP [148,156]. Importantly, acetone (the other ketone body) cannot be biotransformed into acetyl-CoA and ends up eliminated through urine or exhaled [157,158].

It is well understood that glucose is an essential contributor for the precise energetic balance in VSM [159]. However, in 1981, Chace et al. demonstrated in PASM from rabbit that the absence of extracellular glucose increases the oxidation rate of β-HB, highlighting the role of ketones as energy substrates for PASMCs. Moreover, when other substrates such as β-HB, palmitate, leucine, glutamine and isoleucine are present in the external medium, glucose accounts for barely 5% of O_2_ consumption [160]. The authors also found that ketone bodies may supply for the 8–16% of the O_2_ consumption in the VSM. 

High glucose levels may elicit the over production of ROS, mainly through NOX4 activity in endothelial cells [161]. In addition, in these cells, hyperglycemia triggers NF-κB signaling pathway leading to the upregulation of proinflammatory cytokines and endothelial adhesion molecules [162]. The induction of endothelial selectin (E-selectin), intercellular adhesion molecule 1 (ICAM-1), vascular cell adhesion molecule 1 (VCAM-1), and increased mononuclear-endothelial adhesion in addition to ROS generation and NF-κB activity increase vascular permeability and facilitates endothelial barrier dysfunction [162]. Amusingly, red and white wine pomace products (rWPPs and wWPPs) have been shown to reduce the expression of NOX4, thus diminishing ROS production in hyperglycemic endothelial cells [163]. Furthermore, rWPPs and wWPPs improve E-cadherin expression and E-cadherin cell–cell junctions in endothelial cells after INF-γ-induced barrier disruption. Remarkably, these results suggest the potential protective effects of rWPPs and wWPPs in vascular inflammatory ailments where endothelial barrier dysfunction play an essential role [163].

The role of ketones in the development of PH remains elusive. In this regard, the evidence indicates a relationship between the RVF occurring in PH and metabolic disorders (risk factors/comorbidities in human PH), such as high blood sugar [58], insulin resistance [164], dyslipidemia [165], and abdominal obesity [166]. Given these insights and the fact that RVF is the main determining factor in morbidity and mortality in PAH, it has been proposed (and studied) that FA metabolism and its byproducts (ketones) play an important role in RVF and PAH development [167,168]. Hereof, the expression of BDH1 and the oxidation of β-HB are augmented in a model of heart failure, highlight the importance of ketones metabolism in cardiac conditions [169]. Moreover, total blood and myocardial β-HB are increased in patients suffering from heart failure [170].

**Figure 3:**
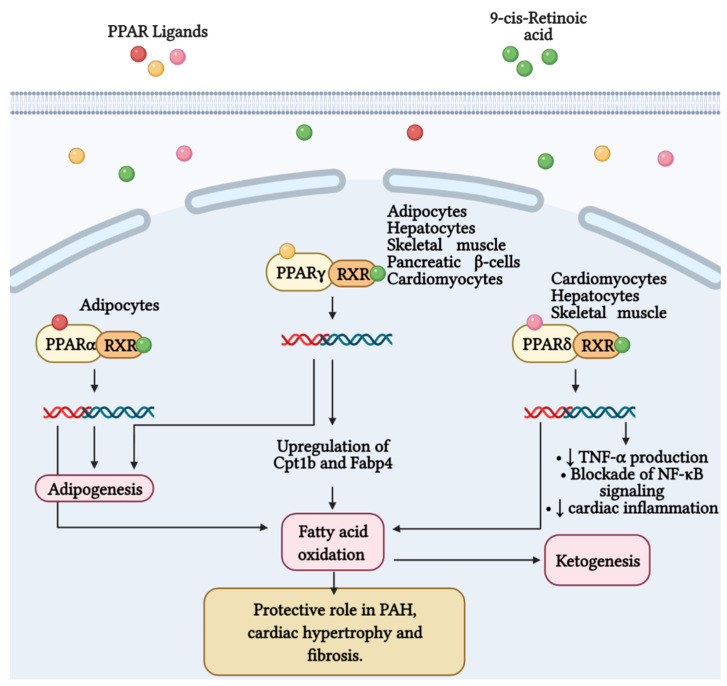
Fatty acid metabolism and its byproducts (ketones) play an important role in the development of right ventricular failure (RVF) and pulmonary arterial hypertension (PAH). Peroxisome proliferator-activated receptors (PPAR) belong to the superfamily of nuclear receptors that serve as ligand-activated transcription factors and consist of three members, PPARα, PPARγ, and PPARδ. These receptors together with retinoid X receptors (RXR) form heterodimers and bind to specific DNA sites to promote genetic transcription. PPARα is a master regulator of adipogenesis expressed mainly in adipose tissue and liver, as is PPARγ. Additionally, PPARγ regulates glucose and fatty acid metabolism in adipocytes, hepatocytes, skeletal muscle, and pancreatic β-cells. PPARγ agonists, such as pioglitazone, increase the expression of Cpt1b and Fabp4, proteins involved in fatty acid oxidation and transport in cardiomyocytes. These effects favor mitochondrial fatty acid oxidation and ATP production, leading to reversal of cardiac hypertrophy, fibrosis, and eliminating severe PAH. Furthermore, PPARδ stimulates fatty acid oxidation, decreases right ventricle hypertrophy and pulmonary congestion. In cardiac inflammation, PPARδ blocks nuclear factor κB (NF-κB) activation and inhibits tumor necrosis factor (TNF)-α synthesis.

It is well known that 70–90% of ATP produced in heart comes from the oxidation of FA in mitochondria. The residual percentage is generated by the metabolism of glucose, ketone bodies and amino acids [171]. In this context, Peroxisome proliferator-activated receptor γ (PPARγ) has been shown to regulate glucose and FA metabolism in adipocytes, hepatocytes, skeletal muscle, and pancreatic β cells [172,173,174]. PPARs belong to the superfamily of nuclear receptors serving as ligand-activated transcription factors. This receptor subfamily is composed of three members, PPARα, PPARγ and PPARδ, which combine with retinoid X receptors (RXRs) forming heterodimers and binding to specific DNA sites to promote genic transcription [173]. PPARγ is a master regulator of adipogenesis expressed mostly in adipose tissue and liver, as well as PPARα. PPARδ is ubiquitously expressed and all three subtypes are expressed in heart [175]. Emerging evidence highlights that PPARγ acts as a strong, protective regulator in PAH [176], PASMCs [177], and PAECs [178]. As it concerns, Legchenko et al., in a SU5416/hypoxia-induced PAH rat model, demonstrated that the oral administration of pioglitazone (a PPARγ agonist) fully abolishes severe PAH and vascular remodeling, and prevents RVF (Figure 3). Moreover, pioglitazone reverses vessel loss, cardiac hypertrophy, and fibrosis, and normalizes glucose uptake. In addition, the authors showed that, the PPARγ agonist augments mitochondrial fatty acids oxidation and ATP production, counteracting the inefficient metabolism of mitochondria characteristic in PAH (Figure 3). To accomplish its function, pioglitazone upregulates the expression of Cpt1b and Fabp4, proteins implicated in the oxidation of FA and their transport in cardiomyocytes, respectively [179]. 

Furthermore, the activity of PPARδ in cardiac metabolism has also been studied. It has been observed that the agonism of this PPAR subtype stimulates fatty acid oxidation [180,181]. Moreover, Ding et al. showed that synthetic ligand of PPARδ (GW0742) abolishes the production of TNFα induced by the stimulation with lipopolysaccharide (LPS) in cardiomyocytes. The authors also found that PPARδ signaling blocks the LPS-induced degradation of IκBs and the subsequent activation of NF-κB. These insights suggest the important role of PPARδ in cardiac inflammation [182]. In addition, genetic ablation of PPARδ in mice leads to the downregulation of essential genes implicated in fatty acids oxidation, highlighting a lipotoxic cardiomyopathy [183]. Most recently, it has been demonstrated that the stimulation of selective PPARδ diminish hypertrophy in the RV and pulmonary congestion in a rodent model of congestive heart failure [184]. 

Emerging evidence indicates that ketone bodies, and in particular β-HB, at low concentrations, potentially contribute to ameliorating endothelial and vascular function in metabolic disease, while elevated concentrations of ketone bodies as observed in diabetic ketoacidosis contribute to the diabetic vasculopathy and diabetic vascular complications. In mammals, β-HB decreases the senescence associated secretory phenotype (SASP) and the senescence of vascular cells. The homeostasis of intracellular Ca^2+^ concentration ([Ca^2+^]_i_) is crucial to maintain the vascular tone. At rest, basal [Ca^2+^]_I_ is tightly regulated to be around 100 nM. After cellular stimulation with a vasoconstrictor agonist, such as norepinephrine, endothelin, vasopressin, etc., [Ca^2+^]_i_ increases, reaching values between 500 nM and 1 mM [128]. 

## 8. Conclusions

Mitochondria are involved in essential cellular regulatory and homeostatic process in the cardiovascular system and particularly in VSMCs. Mitochondrial dysfunction has been widely associated with several diseases including PH. These organelles exert a strict control of ROS and ketones production. Uncontrolled ROS generation and its crosstalk with Ca^2+^ signaling have been shown to contribute and aggravate pulmonary hypertension. Thus, anti-ROS therapies targeting implicated proteins such as RISP should be investigated as novel alternatives for the treatment of this ailment. On the other hand, ketone bodies seem to offer a protection against oxidative stress damage in vitro and in vivo; however, they also may exert anti-inflammatory or pro-inflammatory roles in the cardiovascular system, and further research is needed to understand their different roles. Collectively, a better comprehension of the unique roles of RISP-dependent mitochondrial ROS and their specific interactions with mitochondrial Ca^2+^ signaling, NF-κB-mediated inflammatory responses, and ketone-associated oxidative stress can significantly improve our understanding of the molecular pathogenesis of PH and associated RVF. This new knowledge may also help to develop and implement innovative therapies in the treatment of PH and other vascular diseases.

## Figures and Tables

**Table 1:** WHO classification of pulmonary hypertension (PH).

WHO Group	Clinical Classification	Subtypes
I	Pulmonary arterial hypertension (PAH)	Idiopathic; Drug and toxin-Induced; Heritable; Associated with connective tissue diseases, HIV infection, portal hypertension, schistosomiasis; PAH responder to Ca^2+^ channel blockers; Associated with pulmonary venous/capillaries occlusion; Persistent pulmonary hypertension of the newborn.
II	PH due to left heart diseases	Heart failure; Valvular heart disease; Congenital or acquired cardiomyopathies; Failure with preserved/reduced ejection fraction.
III	PH due to lung disease or hypoxia	COPD/hypoxia that includes COPD; Restrictive lung disease; Pulmonary disease with obstructive and restrictive pattern; Interstitial lung disease; Hypoxia without other lung diseases.
IV	PH due to the obstruction of pulmonary artery	Chronic thromboembolic pulmonary hypertension (CTEPH); Other pulmonary artery obstructions.
V	PH due to unclear/multifactorial mechanisms	Hematologic disorders; Metabolic disorders; Others.

## Abbreviations

AcAC—acetoacetate; ACE—angiotensin-converting enzyme; Ang II—Angiotensin II; AT1—angiotensin II type 1 receptor; ATP—adenosine triphosphate; βHB—beta-hydroxybutyrate; BK_Ca_—large conductance K^+^ channel; BMPRII—bone morphogenetic protein receptor type II; CAT—catalase; [Ca^2+^]_i_—intracellular Ca^2+^ concentration; Ca_V_—voltage dependent Ca^2+^ channel; CH—chronic hypoxia; CoQ—Coenzyme Q/ubiquinol-cytochrome-c reductase; Cyt—cytochrome; DAG—1,2-diacylglycerol; DNA—deoxyribonucleic acid; EC—endothelial cell; ER—endoplasmic reticulum; ET-1 endothelin-1; ETC—electron transport chain; FAD—flavin adenine dinucleotide; FADH—flavin adenine dinucleotide semiquinone; FADH2—flavin adenine dinucleotide hydroquinone form; FKBP12.6—FK506 binding protein 12.6; FOXO3A—forkhead box O3; GPCR—G protein coupled receptor; HCAR2—hydroxy-carboxylic acid receptor 2; HDAC—class I histone deacetylases; HIF-1—hypoxia-inducible factor 1; HPV—hypoxia-induced pulmonary vasoconstriction; HUVEC—human umbilical vein endothelial cells; ICAM-1—intercellular adhesion molecule 1; IH—intermittent hypoxia; IPAH—idiopathic pulmonary arterial hypertension; IP_3_—Inositol 1,4,5-trisphosphate; IP_3_R—Inositol 1,4,5-trisphosphate (receptor); LDL—low density lipoprotein; MCP-1—Monocyte chemoattractant protein; SOD—superoxide dismutase; KD—ketogenic diet; MAM—mitochondrial associated membrane; MAPK—mitogen activated protein kinase; MCU—mitochondrial Ca^2+^ uniporter; mitROS—mitochondrial reactive oxygen species; NADH—nicotinamide adenine dinucleotide; NADPH—nicotinamide adenine dinucleotide phosphate; NF-κB—nuclear factor kappa B; NOX—NADPH oxidase; OXPHOS—oxidative phosphorylation; PAH—pulmonary artery hypertension; PAEC—pulmonary artery endothelial cell; PASM—pulmonary artery smooth muscle; PASMC—pulmonary artery smooth muscle cell; PDK1—gene coding for pyruvate dehydrogenase kinase 1; PDTC—pyrrolidine dithiocarbamate; PG—prostaglandin; PHD—prolyl hydroxylase; PKCε—protein kinase C-epsilon; PLC—phospholipase C; PPARγ—peroxisome proliferator activated receptor-gamma; PPARδ—peroxisome proliferator activated receptor-delta; PVR—pulmonary vascular remodeling; RISP—Rieske iron–sulfur protein; ROS—reactive oxygen species; [ROS]_i_—intracellular ROS concentration; RVF—right ventricular failure; RyR—ryanodine receptor; SR—sarcoplasmic reticulum; TCA—tricarboxylic acid; TNF-α—tumor necrosis factor-alpha; TRP—transient receptor potential channels; UCP—mitochondrial uncoupling protein; VCAM-1—vascular cell adhesion protein-1; VDCC—voltage dependent Ca^2+^ channel; VSM—vascular smooth muscle; VSMC—vascular smooth muscle cell.
